# Measurement Methods in the Operation of Ships and Offshore Facilities

**DOI:** 10.3390/s21062159

**Published:** 2021-03-19

**Authors:** Leszek Chybowski, Arkadiusz Tomczak, Maciej Kozak

**Affiliations:** 1 Faculty of Marine Engineering, Maritime University of Szczecin, ul. Willowa 2, 71-656 Szczecin, Poland; 2 Faculty of Navigation, Maritime University of Szczecin, ul. Wały Chrobrego 1-2, 70-500 Szczecin, Poland; a.tomczak@am.szczecin.pl; 3 Faculty of Mechatronics and Electrical Engineering, Maritime University of Szczecin, ul. Willowa 2, 71-656 Szczecin, Poland; m.kozak@am.szczecin.pl

## 1. Introduction

The development of modern measurement methods for ship systems has occurred due to economic changes and increasingly stringent environmental requirements. At the same time, the specificity of ship systems and the conditions in which they work produce very strict requirements for the reliability and accuracy of the measuring systems on ships and offshore facilities. The digitization of many of the processes carried out on ships, oil rigs, platforms, etc., increases their complexity and security requirements. This book is dedicated to research concerning the measurement methods and condition monitoring of marine systems. 

This collection presents the results of research related to the development of modern systems on ships and offshore facilities, with particular emphasis on measuring and assessing processes occurring in ship propulsion systems, ship navigation systems, maritime communications, maritime safety and alarm systems, marine cargo handling equipment, offshore technological systems, etc.

This book addresses all types of sensors and measurement systems designed for ships and offshore facilities. It provides an advanced forum for the science and technology of sensors and measuring systems. Regarding marine systems such as ships and offshore facilities, the scope of the book includes mostly topics associated with physical sensors, remote sensors, smart/intelligent sensors, sensor devices, sensor technologies and applications, signal processing, data fusion, sensor interfaces, human-computer interactions, sensing systems, and localization and object tracking.

## 2. Special Issue Papers

The above-mentioned topics have been included in the following thematic sections. The research undertaken by the authors of the following sections can be grouped according to the type of equipment being analyzed, particularly including articles on ship machinery and navigational equipment. Specific topics are related to broadly-understood measurement issues, as well as condition monitoring and predicting.

### 2.1. Supporting the Ship Navigation

In this section, we present articles on the subject of aiding navigation, maneuvering, and operating ships in various operating conditions.

The first article in this section, “Full-Scale Maneuvering Trials Correction and Motion Modeling Based on Actual Sea and Weather Conditions”, was prepared by B. Mei et al. [1]. The authors propose a novel sea trials correction method for ship maneuvering. In the paper, the wind and wave drift forces were calculated according to the measurement data. The authors used a pattern search algorithm to calculate the adjustment parameters for wind, waves, sea surface currents, etc.

Subsequently, the team of Y. Yang et al. presented an article entitled “An Attitude Prediction Method for Autonomous Recovery Operation of Unmanned Surface Vehicle” [2]. The authors presented results of experimental launch and recovery technology for an unmanned surface vehicle (USV). To improve the launch accuracy and reduce the influence of sea waves, the authors proposed a stacking model of a one-dimensional convolutional neural network and long short-term memory neural network to predict the attitude of the USV. The authors tested the efficiency and effectiveness of the launch and recovery system, which were demonstrated by its successful application in actual environments.

The subject of unmanned surface vehicle control is taken up by T. Szelangiewicz et al. in their article, “Application of Measurement Sensors and Navigation Devices in Experimental Research of the Computer System for the Control of an Unmanned Ship Model” [3]. The main objective of the research presented in the paper was to design and build a prototype computer system with the necessary measurement sensors and navigation devices to autonomously control an unmanned ship model. The authors presented this type of system and verified its operation on open water.

### 2.2. Optimizing the Operation of Ship Machinery

The second section presents issues related to condition monitoring and optimizing the operation of a ship’s onboard propulsion and power generation.

The first article by D. Kim et al. is entitled, “An Ensemble-Based Approach to Anomaly Detection in Marine Engine Sensor Streams for Efficient Condition Monitoring and Analysis” [4]. The authors proposed an unsupervised anomaly detection method using sensor streams from a marine two-stroke diesel engine to detect anomalous system behaviors that may be a sign of system failure. After detecting an anomaly, clustering analysis was conducted on the anomalous observation to examine anomaly patterns.

In turn, in the article by M. Giernalczyk and P. Kaminski entitled, “Assessment of the Propulsion System Operation of the Ships Equipped with the Air Lubrication System” [5], the authors present the results of the measurements of indicators describing the operational effectiveness of an Air Lubrication System installed on a modern passenger ship. This analysis presents some critical observations regarding the efficiency of the system.

Another article prepared by A. Bogdanowicz and T. Kniaziewicz is entitled, “Marine Diesel Engine Exhaust Emissions Measured in Ship’s Dynamic Operating Conditions” [6]. The article deals with the measurement of emissions from marine engines in dynamic states. The authors proposed a measurement methodology using an exhaust gas analyzer with simultaneous recording of the load indicator, engine speed, inclinometer, and global positioning system (GPS) data. A neural network algorithm was used to model the concentrations of ingredients contained in engine exhaust gases during dynamic states. The proposed method enabled the calculation of emissions of the composition of exhaust gases from the marine diesel engine and also the calculation of the route emissions of the tested vessel.

The fourth article in this section by D. Kim et al. is entitled, “Data-Driven Prediction of Vessel Propulsion Power Using Support Vector Regression with Onboard Measurement and Ocean Data” [7]. The authors proposed a data-driven approach to predict the propulsion power of a vessel. In this study, support vector regression (SVR) was used to learn from big data obtained from onboard measurements and the National Oceanic and Atmospheric Administration (NOAA) database.

In turn, the team of S. German-Galkin and D. Tarnapowicz wrote an article entitled, "Energy Optimization of the ‘Shore to Ship’ System—A Universal Power System for Ships at Berth in a Port” [8]. The authors present an analysis of steady-state electromagnetic and energy processes, allowing the determination of the active and reactive power and losses in a shore-to-ship (STS) system. The presented analytical research enables the development of a control algorithm that optimizes the system’s energy efficiency.

### 2.3. Measurements of Ship Machinery Components

This section includes three articles devoted to geometry measurements and quality assessment of machine driveshaft elements.

The first article by L. Chybowski et al. is entitled “Evaluation of Model-Based Control of Reaction Forces at the Supports of Large-Size Crankshafts” [9]. The article discusses a support control automation system employing force sensors to a large-size crankshaft main journal’s flexible support system. The support reaction forces were changed to minimize the crankshaft elastic deflection as a function of the crank angle. The aim of this research was to verify the hypothesis that the mentioned change can be expressed by a monoharmonic model regardless of the crankshaft structure. The authors’ investigations confirmed this hypothesis. It was also shown that an algorithmic approach improved the mathematical model mapping with the reaction forces due to faster and more accurate calculations of the phase shift angle. The verification of the model for crankshafts with different structural designs made it possible to assess how well the model fit the coefficients of determination that were calculated with finite element analysis (FEA).

Another article was written by K. Miądlicki et al., “Remanufacturing System with Chatter Suppression for CNC Turning” [10]. The article presents the concept of a support system for the manufacture of machine spare parts. The operation of the system is based on a reverse-engineering module enabling feature recognition based on a 3D parts scan. Then, a CAD geometrical model is generated, on the basis of which a machining strategy using the CAM system is developed. The operation of the described system was presented using the example of machining parts of the shaft class. The result is a replacement part, the accuracy of which was compared using the iterative closest point algorithm to obtain the root mean square error at the level of the scanner accuracy.

The third article in this group was prepared by K. Nozdrzykowski et al. and is entitled “The Effect of Deflections and Elastic Deformations on Geometrical Deviation and Shape Profile Measurements of Large Crankshafts with Uncontrolled Sup-ports” [11]. This article presents a multi-criteria analysis of the errors that may occur while measuring geometric deviations of crankshafts that require multi-point support. The analysis in the paper confirmed that the currently-used conventional support method—in which the journals of large crankshafts rest on a set of fixed, rigid vee-blocks—significantly limits the detectability of their geometric deviations, especially those of the main journal axes’ positions. Insights into performing practical measurements, which will improve measurement procedures and increase measurement accuracy, were provided.

### 2.4. Non-Destructive Testing of Ship Machinery Components

With regard to ship machinery, articles devoted to diagnosing the condition of elements of ship mechanisms using acoustic emission (AE) signals should be mentioned. There are two articles on this subject in this issue.

The first is an article by L. Kyzioł et al. entitled, “Acoustic Emission and K-S Metric Entropy as Methods for Determining Mechanical Properties of Composite Materials” [12]. Composites are now a common material for the construction of elements of modern ships and ship mechanisms. The article concerns the use of AE and Kolmogorov-Sinai (K-S) signals metric entropy to determine the mechanical properties of composites. The authors showed that the application of a modern testing machine and very high-quality instrumentation to record measurement data using the K-S metric entropy method and an AE signal allows the determination of a material’s transition from an elastic to a plastic phase.

Another article presenting the application of AE signals is an article by M. Kozak et al. entitled, “Identification of Gate Turn-off Thyristor Switching Patterns Using Acoustic Emission Sensors” [13]. In this case, the material was devoted to assessing the condition of electronic components used in the power supply and control systems of ships. The paper presents an introduction and preliminary tests of a method utilizing an acoustic emission sensor that can be used to detect early-stage damages of the gate turn-off thyristor.

In turn, in the article written by S. Drewing and K. Witkowski, “Spectral Analysis of Torsional Vibrations Measured by Optical Sensors, as a Method for Diagnosing Injector Nozzle Coking in Marine Diesel Engines” [14], the authors discussed the possibility to diagnose the coking of a marine diesel engine injector nozzle by performing a spectral analysis of the crankshaft’s torsional vibrations. The authors presented and verified the new method, which enabled the measuring and calculation of torsional vibrations in engine crankshafts.

### 2.5. Supporting Offshore and Inland Water Operations

In the last section, we present selected issues related to dimensioning of the floating offshore objects and measurements performed from the floating systems.

The first article in this section is a paper prepared by G. Stępień et al. entitled, “Dimensioning Method of Floating Offshore Objects by Means of Quasi-Similarity Transformation with Reduced Tolerance Errors” [15]. The article concerns an improvement in the use of sensors supporting the positioning of floating objects. The accurate measurement of the offsets is vital to establish a mathematical relationship between a sensor and vessel common reference point to achieve sufficient accuracy of the survey data. The authors present the method of transformation by similarity with elements of affine transformation, called Q-ST (Quasi-Similarity Transformation). The method was verified in laboratory conditions, as well as in real conditions.

The second article in this section was prepared by K. Pyrchla et al. and is entitled “Analysis of the Dynamic Height Distribution at the Estuary of the Odra River Based on Gravimetric Measurements Acquired with the Use of a Light Survey Boat—A Case Study” [16]. The authors present possible applications of a dynamic gravity meter for determining the dynamic height along a river. The method described in the article can be applied to measurements in all near-zero-depth areas.

## Data Availability

Authors of particular papers published in this Special Issue are responsible for the data presented and they will provide additional information if required.
